# Enhanced production of sulforaphane by exogenous glucoraphanin hydrolysis catalyzed by myrosinase extracted from Chinese flowering cabbage (*Brassica rapa* var. *parachinensis*)

**DOI:** 10.1038/s41598-019-46382-7

**Published:** 2019-07-08

**Authors:** Supakarn Sangkret, Patsaporn Pongmalai, Sakamon Devahastin, Naphaporn Chiewchan

**Affiliations:** 1 Advanced Food Processing Research Laboratory, Department of Food Engineering, Faculty of Engineering, King Mongkut’s University of Technology Thonburi, 126 Pracha u-tid Road, Tungkru, Bangkok 10140 Thailand; 2 The Academy of Science, The Royal Society of Thailand, Dusit, Bangkok 10300 Thailand

**Keywords:** Chemical engineering, Process chemistry

## Abstract

Sulforaphane formation via endogenous route is known to be less effective. Exogenous hydrolysis of the sulforaphane precursor is therefore of interest. Here, myrosinase activity was first determined to identify a suitable source of the enzyme from selected *Brassica* vegetables. Extracted enzyme was then evaluated for its thermal stability to establish a condition for extraction. Chinese flowering cabbage was selected as the source of myrosinase; suitable extraction condition was at 40 °C for 90 min. Enzyme extract was used to hydrolyze glucoraphanin standard into sulforaphane at 30 °C and pH 6. Exogenous hydrolysis reached the equilibrium with the reverse reaction after 30 min; sulforaphane concentration remained unchanged afterward. Molar fractional conversion of glucoraphanin into sulforaphane at 30-min hydrolysis was around 48%. In comparison with exogenous hydrolysis by myrosinase extracted from broccoli, which indeed exhibits higher activity than the enzyme extracted from Chinese flowering cabbage, no conversion of glucoraphanin into sulforaphane was unexpectedly observed.

## Introduction

*Brassica* is a family of vegetables that includes such varieties as broccoli, cauliflower, cabbage and mustard. These vegetables are known to contain glucosinolates (GLSs), which are sulfur- and nitrogen-containing compounds. Different GLSs have different side groups; such differences result in a variety of their biological activities^1–3^.

Upon cellular damage, glucoraphanin, which is a type of GLSs, is hydrolyzed by the enzyme myrosinase, which is released from the plant myrosin cells, into sulforaphane^4^. Sulforaphane is a much desirable compound as it exhibits strong anticarcinogenic activity^5,6^. However, epithiospecifier protein (ESP), which is a myrosinase cofactor, is known to compete with myrosinase and hence impedes the formation of sulforaphane; ESP leads to the formation of sulforaphane nitrile, which is undesirable as it contains no anticarcinogenic activity^7^. Moreover, sulforaphane formation is known to be influenced by the hydrolysis conditions, including pH and temperature^8^. It has indeed been reported that the formation of sulforaphane nitrile is enhanced at a lower pH (pH < 3), whereas the formation of sulforaphane is enhanced at a higher pH (pH 5–7)^9^. Shen *et al*.^10^ also reported that the hydrolysis reaction capability depends on many factors, including enzyme-to-substrate ratio, reaction duration and pH, among other factors. These factors are clearly not easy to control if the reaction is to be taking place endogenously.

To avoid sulforaphane nitrile formation and to obtain a higher hydrolysis yield of sulforaphane, exogenous hydrolysis has been brought into interest. Glucoraphanin and myrosinase could in this case be separately extracted from their respective sources and then later reacted. The reaction conditions could be better controlled, so a higher hydrolysis yield could be expected. Shen *et al*.^10^, for example, reported that exogenous conversion of broccoli-based glucoraphanin into sulforaphane was as high as 78%; this conversion level was much higher than that in the case of endogenous hydrolysis, which was only 35%.

Despite some existing studies on exogenous hydrolysis of glucoraphanin into sulforaphane, there are still some uncertainties on the role of other constituents that may be present in a vegetable extract on the hydrolysis reaction. Glucoraphanin standard should first indeed be used as a benchmark substrate, so that the information on the maximum possible hydrolysis yield can be obtained. This would also allow one to more easily assess the capability of myrosinase extracted from various plant sources to convert the substrate into sulforaphane. It should be noted also that information on the activity and thermal stability of myrosinase extracted from various sources is not yet well established.

In this study, preliminary investigation was first performed to determine and compare the activities of myrosinase from Chinese flowering cabbage and mustard seeds. The former was chosen as it is a widely and inexpensively available vegetable in Thailand, while the latter was chosen as it is reported to be a good source of myrosinase with high thermal stability^11^. Thermal stability of myrosinase from the selected source was then evaluated to establish a condition for the myrosinase extraction process. The enzyme extract was allowed to react with glucoraphanin standard into sulforaphane. Evolutions of both glucoraphanin and sulforaphane concentrations as well as myrosinase activity were monitored during the hydrolysis reaction. The hydrolysis results were finally compared with those in the case where myrosinase extracted from broccoli, which is known to exhibit higher activity, was used.

## Results and Discussion

### Activities of myrosinase from different sources

**Figure 1:**
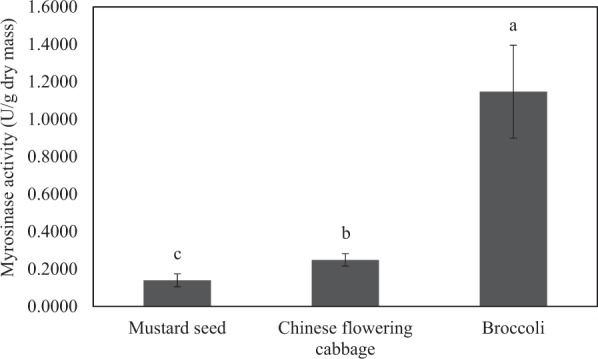
Activities of myrosinase from different sources. Different letters over the bars indicate that values are significantly different (*p* < 0.05).

A preliminary study was first conducted to select a suitable source of myrosinase; the selection was based on myrosinase activity of an extract from each vegetable source. The activities of myrosinase from different sources are shown in Fig. 1. Chinese flowering cabbage and mustard seeds exhibited lower enzyme activities, i.e., 0.2485 and 0.1394 U/g dry mass, respectively. Myrosinase of the highest activity was from broccoli; the activity was noted to be 1.1470 U/g dry mass. Comparing with mustard seeds, Chinese flowering cabbage is an obvious choice for all subsequent hydrolysis experiments. Chinese flowering cabbage was also a more preferred source of myrosinase to broccoli as the enzyme from the former vegetable exhibits some superior characteristics to that from the latter, as will be shown in the later section.

### Thermal stability of myrosinase from Chinese flowering cabbage

After Chinese flowering cabbage had been selected as the source of myrosinase, determination of its thermal stability was conducted. Such an information is important to avoid the condition that might inactivate myrosinase during the enzyme extraction process.

**Figure 2:**
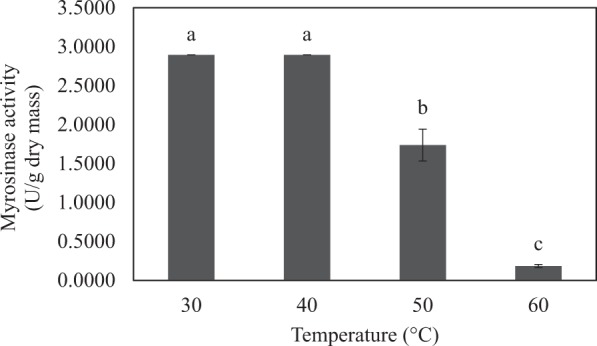
Activity of myrosinase from Chinese flowering cabbage as a function of temperature. Different letters over the bars indicate that values are significantly different (*p* < 0.05).

The results on myrosinase activity as a function of temperature are shown in Fig. 2. Myrosinase activity increased to its maximum value, which was 2.8940 U/g dry mass, when the sample temperatures were 30 and 40 °C. When the temperature increased beyond 40 °C, however, the enzyme activity decreased. The decreased activity is due to the fact that myrosinase is a proteinaceous molecule made of folded amino acids^12,13^. When the enzyme is subject to a higher temperature than its denaturation temperature (around 40 °C in this case), the enzyme structure would change from folded (natural state) to unfolded (denatured state). This results in turn in the inability of enzyme to function and hence the decreased measured activity. Based on the previously reported results for mustard seeds^11,14^, myrosinase activity was noted to decrease when the temperature of the experimental system is higher than 60 °C. Although mustard-seeds myrosinase is more thermally tolerant, its activity was noted to be lower than that of the enzyme from Chinese flowering cabbage.

Based on the above-mentioned results, the condition for myrosinase extraction from Chinese flowering cabbage must be selected in such a way that the temperature of the sample would never exceed 40 °C.

### Optimal condition for myrosinase extraction from Chinese flowering cabbage

In the first set of experiments, the recommended methods of Pongmalai *et al*.^15^ were used to extract myrosinase from Chinese flowering cabbage. Thirty-mM citrate/phosphate buffer at pH 7 was used as the extraction solvent. The extract turned out to possess low myrosinase activity; the value was only 0.0143 U/g dry mass. When such an extract was used to perform exogenous hydrolysis of the glucoraphanin standard, no sulforaphane was formed; the glucoraphanin concentration remained unchanged. In such a case, the ratio of substrate to enzyme seemed inappropriate. An extract with a higher enzyme concentration or activity was needed. Similar observation has indeed been reported. Montilha *et al*.^16^, for example, stated that the substrate-to-enzyme ratio had a significant effect on enzymatic hydrolysis of okara protein and hence the formation of the protein hydrolysate.

**Figure 3:**
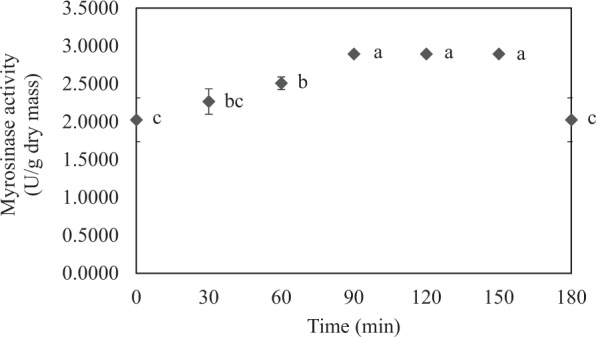
Evolution of myrosinase activity from Chinese flowering cabbage during extraction at 40 mbar and 40 °C. Different letters at different extraction times indicate that values are significantly different (*p* < 0.05).

To obtain an enzyme extract with higher myrosinase concentration/activity, an alternative extraction process, i.e., solvent extraction under vacuum, was attempted. Extraction was in this case conducted at a pressure of 40 mbar and a temperature of 40 °C. Due to the use of vacuum, solvent (DI water) boiled at a lower temperature. Such a boiling led to more extensive disruption of the vegetable cells and hence better release of the cellular contents, including myrosinase. Since the extraction was conducted at a lower temperature, the extracted myrosinase was not thermally damaged. Moreover, since DI water was used as the extraction solvent instead of citrate/phosphate buffer, this alternative extraction method can be considered to be greener. Evolution of the myrosinase activity during this alternative extraction process is shown in Fig. 3.

It can be seen in Fig. 3 that increasing the extraction time (but only to a certain point) resulted in an extract with increased myrosinase activity. The highest enzyme activity (of 2.8941 U/g dry mass) was noted when the extraction time was 90–150 min; the activity significantly (*p* < 0.05) dropped when the extraction time was extended to 180 min. The reason for the reduced activity is that enzyme denaturation is a function of both time and temperature^17,18^. Extended extraction time might have resulted in the damage of non-covalent bonds, which maintain the three-dimensional structure of enzyme^19,20^. This led in turn to the loss of its catalytic activity. The optimal extraction time was selected as 90 min as this is the shortest time to obtain an extract with the highest myrosinase activity.

### Exogenous hydrolysis of glucoraphanin by myrosinase extracted from Chinese flowering cabbage

**Figure 4:**
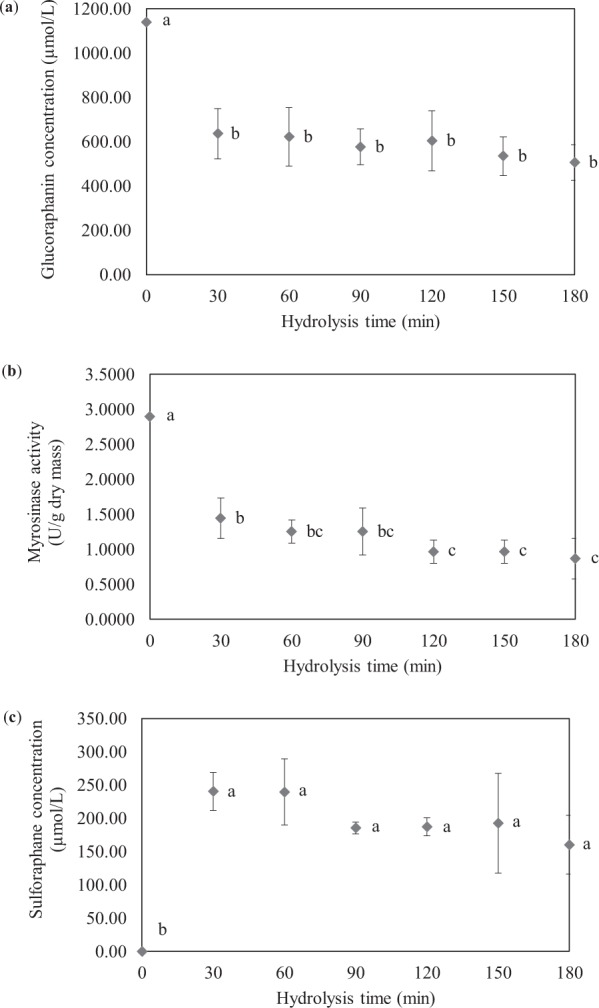
Evolutions of bioactive compounds concentrations and activity of myrosinase from Chinese flowering cabbage during exogenous hydrolysis. (**a**) Glucoraphanin concentration; (**b**) Myrosinase activity and (**c**) sulforaphane concentration. Different letters at different hydrolysis times indicate that values are significantly different (*p* < 0.05).

Evolutions of the glucoraphanin and sulforaphane concentrations as well as myrosinase activity during the hydrolysis reaction are illustrated in Fig. 4. As mentioned in the Materials and Methods section, glucoraphanin standard was prepared at a concentration of 1,139.45 µmol/L (500 ppm); this was noted as the initial glucoraphanin concentration of the reaction mixture. The initial activity of myrosinase extracted from Chinese flowering cabbage was at 2.8940 U/g dry mass. As expected, the initial sulforaphane concentration was null.

Upon the hydrolysis reaction, the concentration of glucoraphanin decreased to 636.39 µmol/L at 30 min, while myrosinase activity decreased by half compared to the initial value, to 1.4470 U/g dry mass. The hydrolysis reaction led to the formation of sulforaphane; the concentration of this compound increased to 240.33 µmol/L. After 30 min, the concentration of glucoraphanin remained unchanged (*p* > 0.05). This is probably due to the fact that the hydrolysis reaction of interest is a rapid one. The remaining substrate might also not be present at an adequate concentration for the hydrolysis reaction to significantly proceed. Another possible reason for the limited consumption of glucoraphanin is the limited availability of the enzyme. The situation reported here is quite different from the case of endogenous hydrolysis where the reaction proceeds at a much lower rate. In such a case, substrate and enzyme are most of the time not in direct contact and transport of substrate and/or enzyme needs to take place before the reaction can occur. This results in a slower reaction compared to that taking place exogenously. The observation here agrees well with that of Shen *et al*.^10^ who reported that exogenous hydrolysis of GLSs required shorter time than its endogenous counterpart.

Myrosinase activity slightly but insignificantly (*p* > 0.05) decreased during 30 to 90 min of the reaction. After 90 min, the activity again slightly decreased; the activity remained unchanged after 120 min of the reaction. The decrease in the myrosinase activity might be due to the fact that crude (and not pure) enzyme extract was used. Such an extract might contain other compounds that might have competed with glucoraphanin for active sites of myrosinase, resulting in more stable complexes that prevented further hydrolysis of glucoraphanin^21^. Note that in this study, sinigrin was used as the substrate during the quantification of myrosinase activity. Increasing the hydrolysis time might simply lead to the formation of other complexes rather than sinigrin-myrosinase complexes. This resulted in the decrease in the quantified myrosinase activity. After 30 min of the reaction, the concentration of sulforaphane remained unchanged (*p* > 0.05). The reason for this observed phenomenon is probably the same as that mentioned earlier for the glucoraphanin concentration evolution.

After 3 h of the hydrolysis time, glucoraphanin concentration remained at 506.93 µmol/L, while the sulforaphane concentration was 160.17 µmol/L; concentration of the latter did not increase any further although the myrosinase activity still remained at 0.8682 U/g dry mass. It is possible to conclude in this case that either the substrate concentration or enzyme activity dropped to an inadequate value after 30 min; such a lower value was not sufficient for the formation of sulforaphane to take place.

If only the first 30 min of the reaction where the myrosinase activity decreased by half compared to its initial value and the glucoraphanin concentration was around 500 µmol/L and the sulforaphane concentration was around 240 µmol/L was considered, the molar fractional conversion of glucoraphanin into sulforaphane was calculated to be around 48%; this conversion value is lower than that reported by Shen *et al*.^10^ who mentioned that exogenous hydrolysis reaction of glucoraphanin into sulforaphane was around 78%. Since glucoraphanin concentration and substrate-to-enzyme ratio in our study were lower than those used by Shen *et al*.^10^, lower conversion of glucoraphanin into sulforaphane is not unexpected.

### Exogenous hydrolysis of glucoraphanin by myrosinase extracted from broccoli

The results on exogenous hydrolysis of glucoraphanin by myrosinase extracted from Chinese flowering cabbage were compared with those by myrosinase extracted from broccoli. This was done since broccoli-based myrosinase is known to exhibit higher activity. It is therefore of interest to see if sulforaphane could be more extensively formed when using such a seemingly better enzyme to catalyze the reaction.

First of all, an optimal time at the same pressure and temperature of 40 mbar and 40 °C for myrosinase extraction from broccoli was determined. Myrosinase activity reached its maximum value of 13.0235 U/g dry mass at the extraction time of 150 min (data not shown). This activity value was expectedly higher than that of myrosinase from Chinese flowering cabbage of 2.8941 U/g dry mass.

**Figure 5:**
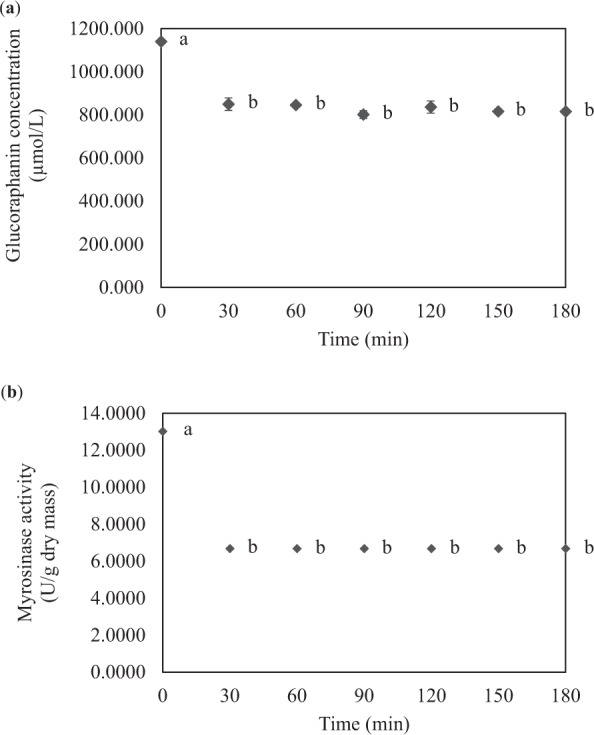
Evolutions of glucoraphanin concentration and activity of myrosinase from broccoli during exogenous hydrolysis. (**a**) Glucoraphanin concentration and (**b**) myrosinase activity. Different letters at different hydrolysis times indicate that values are significantly different (*p* < 0.05).

Figure 5 shows the evolutions of glucoraphanin concentration and broccoli-based myrosinase activity during exogenous hydrolysis at the same 30 °C and pH 6. Glucoraphanin concentration decreased to 849.280 µmol/L after 30 min of the hydrolysis. The concentration of this standard compound then remained almost unchanged. Likewise, the myrosinase activity decreased after 30 min of the hydrolysis and then remained constant. Interestingly and unexpectedly, no sulforaphane formation during the whole course of the hydrolysis reaction was observed. It has indeed been reported that sulforaphane nitrile could be formed when broccoli is crushed; this compound is presented as the predominant glucoraphanin breakdown product instead of sulforaphane^9^. Formation of complexes in crude enzyme extract with glucoraphanin as discussed earlier might also be another reason for the lack of sulforaphane formation but to the decrease in the glucoraphanin concentration and myrosinase activity during an earlier period of the hydrolysis reaction. This may imply that myrosinase from broccoli exhibits lower selectivity toward glucoraphanin than that from Chinese flowering cabbage. Note that selectivity of an enzyme is related to the size and shape of its active sites^22^. Enzyme from different sources may have different structures of active sites and hence different selectivities. Cohen *et al*.^23^, for example, observed the hydrolysis of carboxymethylcellulose (CMC) by endoglucanases from six different cell cultures and found that although endoglucanase Tr Cel 45a exhibited poor hydrolysis efficiency, such an enzyme could yield the most diverse mixture of soluble sugars in comparison with the enzymes from other cultures. Note that no information on the selectivity of myrosinase from different sources such as that reported in our study is so far available. Using enzyme (in particular, crude enzyme extract) of higher activity does not always guarantee better hydrolysis results.

## Conclusion

Information on the activities of myrosinase from selected *Brassica* vegetable sources and on the feasible use of the selected myrosinase extract was obtained and used as a guideline to enhance the production of sulforaphane from glucoraphanin. Chinese flowering cabbage was selected as the source of myrosinase. Optimal condition to obtain extract with the highest myrosinase activity was the pressure of 40 mbar and temperature of 40 °C for 90 min. Upon hydrolysis for 30 min, molar fractional conversion of glucoraphanin into sulforaphane was 48%. For comparison purpose, exogenous hydrolysis of glucoraphanin by myrosinase extracted from broccoli was also conducted. Interestingly, no sulforaphane formation unexpectedly took place, probably due to the formation of sulforaphane nitrile. Apart from the possibly lower selectivity of the broccoli-based myrosinase, formation of complexes in crude enzyme extract with glucoraphanin might also be the reason for the lack of sulforaphane formation. Determination of extraneous compounds in crude extract and their roles in preventing the formation of sulforaphane should be conducted to better understand the behavior of the GLSs-myrosinase system.

## Materials and Methods

### Materials

Fresh Chinese flowering cabbage (*Brassica rapa* var*. parachinensis*) and broccoli (*Brassica oleracea* var*. italica*) were bought from Thrungkru 61 market (Bangkok, Thailand). The vegetables were used for an experiment within the day of their purchase. White mustard seeds (*Sinapis alba* L.) were purchased from Tone Brothers Inc. (Ankeny, IA) and kept at room temperature.

Prior to the start of each experiment, vegetables were washed with water and drain to get rid of excess water. A stainless-steel knife was used to cut only the leaves of Chinese flowering cabbage and florets of broccoli. Each of these vegetable parts were then chopped with an electric chopper (Moulinex, DPA141, Ecully, France) for 1 min to obtain an average size of 2–3.5 mm. Mustard seeds were ground with a blender (Waring, 32BL80, New Hartford, CT) for 1 min to obtain an average size of 0.2–0.5 mm.

### Chemicals

Glucoraphanin potassium salt was purchased from Chromadex (Irvine, CA). Sinigrin hydrate and sulforaphane were provided by Sigma-Aldrich (St. Louis, MO). Citric acid, disodium hydrogen phosphate, ethylene diamine tetra acetic acid (EDTA), dimethyl sulfoxide (DMSO), glacial acetic acid, ammonium acetate, HPLC-grade methanol and HPLC-grade acetonitrile were purchased from RCI Labscan (Bangkok, Thailand), while HPLC-grade water was supplied by Sithiporn (Bangkok, Thailand).

### Determination of myrosinase activity

#### Preparation of enzyme extract

Following the methods of Pongmalai *et al*.^15^ with some modification, 2 g (dry mass) of each *Brassica* sample was blended with 50 mL of 30-mM citrate/phosphate buffer (pH 7) for 1 min. The mixture was filtered through a 425-µm stainless steel screen. The filtrate was centrifuged at 30,000 × g and 4 °C for 4 min before being filtered through Whatman No. 1 filter paper. Supernatant was determined for the myrosinase activity.

#### Quantification of myrosinase activity

Fifty µL of the above-mentioned supernatant, 1.35 mL of 32.22-mM citrate/phosphate buffer (pH 6.5) with 1.07-mM EDTA and 100 µL of 37.50-mM sinigrin were mixed at 30 °C as a sample solution. One-hundred µL of DI water was used instead of sinigrin solution to prepare the reference solution. Both the sample and reference solutions were placed into a pair of 1.5-mL quartz glass cuvettes (Hellma Analytics, Müllheim, Germany). The absorbance was measured via the use of a spectrophotometer (Shimadzu, UV-2600, Kyoto, Japan) at 227 nm and 30 °C at every 5-s interval for 5 min. Myrosinase activity was calculated from the rate of decrease in the absorbance of sinigrin in the reaction mixture. A unit of myrosinase activity (U) refers to the hydrolysis of one μmol of sinigrin/min.

#### Determination of thermal stability of myrosinase

Thermal stability of myrosinase was determined as per the recommended methods of Yen and Wei^24^ with some modification. Five g of chopped sample (only Chinese flowering cabbage in this case) was placed in a pouch and immediately vacuum sealed. Four sample pouches were prepared; each pouch was heated at 30, 40, 50 or 60 °C for 30 min. After heating, the pouch content was mixed with 500 mL of DI water. The mixture was then placed in a 1,000-mL flask. Extraction was conducted at 40 °C and 40 mbar for 90 min; choices of the extraction temperature and time were as mentioned in the Results and Discussion section. The resulting content was filtered through a 425-µm stainless steel screen and through Whatman No. 1 filter paper. Supernatant was determined for the myrosinase activity as per the methods described earlier.

#### Determination of optimal extraction condition for myrosinase

Fifty g of chopped Chinese flowering cabbage was mixed with 500 mL of DI water in the Waring blender for 1 min. The mixture was then placed in a 1,000-mL flask; extraction was conducted at 40 °C and 40 mbar for up to 3 h. A 25-g sample was taken at every 30-min interval, filtered through a 425-µm stainless steel screen and then through Whatman No. 1 filter paper. Supernatant was determined for the myrosinase activity as per the methods described earlier. Optimal extraction time (90 min) was selected as the time that led to an extract with the highest myrosinase activity.

#### Exogenous hydrolysis of glucoraphanin

Glucoraphanin standard at a concentration of 1,139.45 µmol/L (or 500 ppm) was used as the substrate. The selected concentration was based on the results of our preliminary study, which revealed that the maximum glucoraphanin concentration extractable from a typical Thai vegetable was around 500 ppm.

Two hundred µL of the enzyme extract along with the glucoraphanin standard was added to a microcentrifuge tube; the content was mixed for 30 s. The reaction temperature and pH were maintained at 30 °C and pH 6, respectively. Hydrolysis was conducted for different periods of time in different tubes; the reaction time was 0, 30, 60, 90, 120, 150 and 180 min. At each pre-determined sampling time, a sample was collected to monitor the changes in the glucoraphanin and sulforaphane concentrations as well as myrosinase activity.

#### Determination of glucoraphanin concentration

Each hydrolysis sample was analyzed following the methods of Pongmalai *et al*.^15^. Ten µL of the sample was injected into Xselect CSH C_18_ HPLC column (4.6 × 25  mm) (Waters, Milford, MA). 30% of HPLC-grade methanol with 70% of 30-mM ammonium acetate buffer (pH 5) was used as the mobile phase; the flow rate was set at 1 mL/min. Detection of glucoraphanin was conducted at 233 nm via the use of an UV detector (Waters, 2998, Milford, MA). A standard calibration curve was prepared using glucoraphanin potassium salt in HPLC-grade water at concentrations of 0–1,000 μg/mL.

#### Determination of sulforaphane concentration

One hundred µL of each hydrolysis sample was placed in a 50-mL flask and evaporated at 50 °C and 23 mbar for 5 min. One hundred µL of DMSO was then added; the mixture was kept in a vial at −18 °C until the time of the analysis, which was again conducted following the methods of Pongmalai *et al*.^15^. Ten µL of the sample was injected into Xselect CSH C_18_ HPLC column (4.6 × 25  mm) (Waters, Milford, MA). 15% of HPLC-grade acetronitrile with 85% of 1% (v/v) acetic acid was used as the mobile phase at a flow rate of 1.2 mL/min. UV detector at a wavelength of 254 nm was used to detect sulforaphane. The sulforaphane concentration was calculated from the standard curve of sulforaphane, which was prepared by using DMSO as a solvent at concentrations of 0–200 µg/mL.

### Statistical analysis

All the experiments were performed in triplicate and the results are reported as means ± standard deviations. The experimental data were subject to the analysis of variance (ANOVA). Differences of the means were established using Duncan’s new multiple range tests at a confidence level of 95%. All statistical calculations were performed using SPSS® software (version 16, SPSS Inc., Chicago, IL).

## Data Availability

Experimental and relevant data are available from the authors upon request.
